# Research on the Influence of Thermoplastic Extrusion Parameters and Annealing Heat Treatment on the Compressive Strength of Specimens Made from PETG and Recycled PETG

**DOI:** 10.3390/polym18101201

**Published:** 2026-05-14

**Authors:** Dragos Gabriel Zisopol, Mihail Minescu, Dragos Valentin Iacob

**Affiliations:** 1 Mechanical Engineering Department, Petroleum-Gas University of Ploiesti, 100680 Ploiesti, Romania; zisopold@upg-ploiesti.ro (D.G.Z.); mminescu@upg-ploiesti.ro (M.M.); 2 Department of Mechanical Engineering, Doctoral School, Petroleum-Gas University of Ploiesti, 100680 Ploiesti, Romania

**Keywords:** additive manufacturing, thermoplastic extrusion, experimental determinations, optimization, heat treatment

## Abstract

This paper presents the results of research conducted on the influence of thermoplastic extrusion parameters (layer height per pass—L_h_ and the percentage fill density—I_d_) and heat treatment (annealing) on the compressive strength of specimens manufactured by thermoplastic extrusion of virgin and recycled polyethylene terephthalate glycol (PETG and rPETG) filaments. To support the study, using the parameters L_h_ = (0.10–0.20) mm and I_d_ = (50–100)%, 90 compression test specimens were manufactured from PETG and rPETG (45 specimens for each material), which were subsequently subjected to heat treatment by annealing at a temperature of 75 °C for a period of 180 min. The results obtained highlight a significant correlation between the variable manufacturing parameters (L_h_ and I_d_) and the compressive strengths (C_s_). The average compressive strengths of the 45 specimens made from PETG are 44.15% lower than the average compressive strengths of the specimens made from rPETG. The annealing heat treatment resulted in a 31.40% decrease in the average compressive strengths of the specimens made from PETG and a 0.63% increase in the average compressive strengths of the specimens made from rPETG. The specimens made from PETG exhibited increased thermal sensitivity, which led to molecular relaxation, while rPETG exhibited superior thermal stability acquired through recycling.

## 1. Introduction

Additive technologies have marked industrial engineering due to the paradigm shift through which they have overcome the limitations of conventional manufacturing technologies—subtractive and formative [1,2,3]. Unlike conventional manufacturing technologies, additive manufacturing technologies stand out due to the significantly higher efficiency of material use and profitability for small-scale production [4,5,6,7]. The additive manufacturing process consists of making objects by successively depositing layers of material according to the work instructions in the G-Code file until the part is completely made [8,9,10]. The range of additive technologies is diverse; currently, within the ISO/ASTM 52900:2021 standard (Additive manufacturing—General principles—Fundamentals and vocabulary, Geneva, Switzerland, 2021), seven categories of additive technologies are regulated, which are based on photopolymerization in the tank, powder melting, material extrusion, material jet spraying, binder spraying, direct energy deposition, and sheet lamination [11,12,13,14,15,16]. All seven categories are representative of additive manufacturing technologies and their applicability in industries such as defense, automotive, aerospace, medical, oil and gas [17,18,19].

One of the most appreciated additive technologies is that based on thermoplastic extrusion (FDM). This technology is based on melting a thermoplastic, generally a filament, which is then extruded and deposited initially on the machine platform and later on top of the previously deposited layer [20,21,22]. Additive manufacturing via thermoplastic extrusion is popular due to its accessibility and versatility, as well as the diversified range of equipment and materials. However, despite the versatility of this additive manufacturing technology, there are also limitations that are generally represented by the resistance of the manufactured parts [23,24,25]. There is particular interest among researchers in increasing the technical performance of parts manufactured additively using thermoplastic extrusion. In this context, a proposed solution to improve the technical characteristics is the application of thermal treatments. In the study presented by the authors in [26], it is demonstrated that the application of annealing heat treatment to 3-point bending parts resulted in a 17% increase in bending strength. In the study presented in [27], the authors highlight the importance of optimizing heat treatment parameters based on the type of material. The study investigates the impact of the layer height deposited in one pass and the annealing heat treatment parameters on the tensile strengths and dimensional accuracy of specimens manufactured by thermoplastic extrusion of polylactic acid (PLA), polyethylene terephthalate glycol (PETG) and polyethylene terephthalate glycol reinforced with carbon fibers (PETGCF). The conclusions of the study show that the layer height deposited in one pass and the duration of the heat treatment cycle significantly influence the tensile strengths of PLA, PETG, and PETGCF specimens. The dimensional accuracy of the heat-treated tensile specimens is good, with the deviation from the initial measurements being a maximum of 5%. In the work [28], the effects of annealing heat treatment on the mechanical properties of 3D-printed composites (PETG-CF, PETG-KF) are studied. The results of the study show that heat treatment improves the mechanical performance of the parts by up to 20%. Compared to the non-heat-treated parts, the heat-treated parts achieved flexural strengths that were 10.20–31.8% higher, and the maximum flexural modulus was 17.60–62.2% higher. The study presented in the work [29] investigates the impact of annealing and load annealing heat treatment on the tensile strength, ductility and dimensional stability of parts additively manufactured by thermoplastic extrusion from ABS. Annealing under load consists of applying an additional weight to the specimens. The results of the study show that applying the annealing heat treatment at a temperature below the glass transition temperature causes a 7.6% increase in tensile strength, and increasing the annealing temperature increases ductility by up to 17.56%. In the work [30], the effects of annealing heat treatment and salt remelting on the tensile behavior of specimens additively manufactured from PLA and PETG are examined. The results of the study show that the annealing heat treatment performed for one hour at a temperature of 60 °C led to a 24% increase in the tensile strength of PLA specimens, although the parts suffered major deformation. The salt remelting process is noted for improving the tensile strength of parts manufactured from PETG and for maintaining high dimensional accuracy. The paper [31] presents the results of research on optimizing the mechanical properties of acrylonitrile styrene acrylate (ASA) using a hybrid simulated annealing approach with TOPSIS (Technique for Order Preference by Similarity to Ideal Solution). The study results show that the best mechanical characteristics were obtained for specimens manufactured with a deposited layer height of 0.10 mm, an extrusion temperature of 260 °C, a platform temperature of 90 °C, a printing speed of 37 mm/s, a filling density of 70%, 6 contours, and a 0° orientation. The paper [32] focuses on the analysis and optimization of the annealing heat treatment for the manufacture of PETG tensile parts. The study varied the filling percentage, treatment temperature, and time. The conclusions of the study show that the optimal parameters are a 100% filling percentage, a temperature of 90 °C, and a treatment time of 20 min. The paper [33] presents the results of a study on the effects of heat treatment on the properties of parts made of PEEK reinforced with short and continuous fibers. The study demonstrates that the annealing treatment improves the crystallinity of the material and the adhesion between layers, resulting in improved tensile and bending properties. The paper [34] shows that by performing the annealing heat treatment at t = 100 °C for a duration of 4 h, the tensile strength of additively manufactured PLA parts increases by up to 80%. In the paper [35], the importance of homogeneous heating conditions and the heating effect on the mechanical characteristics of parts made additively by thermoplastic extrusion was investigated. The results of the study show that by reducing the height of the deposited layer, increasing the heating time and applying an external pressure, the voids inside the parts are reduced. The findings of the study [36] show that the preheating temperature helps in eliminating lateral cracks, and post-treatment significantly improves molecular diffusion and crystallization. In paper [37], the authors present a study on the development of a new post-processing technique for parts manufactured using thermoplastic extrusion, aiming to improve surface roughness and reduce heat absorption. The post-processing technique used in the study improved surface roughness by 2.06 µm. The study in paper [38] investigates the influence of the parameters of thermoplastic extrusion and heat treatment on the mechanical properties of parts manufactured from PLA. The conclusions show that using a heat treatment temperature above the transition temperature leads to improvements in mechanical characteristics. In paper [39], the effects of heat treatment on the mechanical properties of additively manufactured parts from PLA are studied. The results of the study show that heat treatment caused deformation of the parts and improved the mechanical characteristics by 4.88–10.26%. Paper [40] presents the results of a study on the influence of extrusion temperature and annealing heat treatment on the mechanical properties of additively manufactured parts from PLA. The conclusions of the study show that by increasing the extrusion temperature, the adhesion between the layers is improved, and the annealing heat treatment increases the percentage of crystallinity, which leads to improved mechanical characteristics. Paper [41] presents the results of research on the influence of heat treatment on the crystallinity and mechanical properties of additively manufactured parts from PLA. The conclusions of the study show that heating the samples above the glass transition temperature increases crystallinity for all samples. In studies [42,43,44,45,46,47,48,49], research is presented that aims to transition to a circular economy in additive manufacturing through thermoplastic extrusion.

Considering the current research in the literature and the studies in the bibliographic references analyzed, research opportunities that have not yet been exploited have been identified: the influence of annealing heat treatment on the compressive behavior of additively manufactured PETG specimens in both virgin and recycled forms. In this context, this work investigates the influence of thermoplastic extrusion parameters (layer height deposited per pass—L_h_ and percentage filling density—I_d_) and annealing heat treatment on the compressive strengths of additively manufactured parts made of PETG and recycled PETG (rPETG). The novelty of this work lies in the use of recycled PETG filament and the heat treatment parameters.

**Figure 1:**
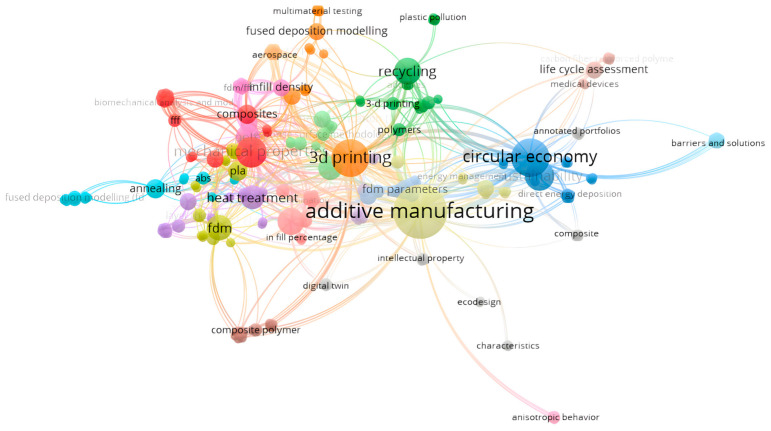
The co-occurrence network of keywords within the studied bibliography.

Using the database from the studied bibliography and VOS Viewer software version 1.6.20, the map in Figure 1 was generated, representing the co-occurrence of keywords in the studied works and the links between them [50].

## 2. Materials and Methods

### 2.1. Additive Manufacturing of Compression Specimens by Thermoplastic Extrusion of PETG and rPETG Filaments

Additive manufacturing is based on a 3D model that is converted to Standard Triangle Language (STL) format and subsequently processed in slicing software version 1.2.4, where the printing parameters are set. This software transforms the information regarding the part profiles and printing parameters into work instructions in G-Code format. For this study, the 3D model of the compression specimen was designed in SolidWorks 2023 software, and the model was subsequently converted to STL format [51].

**Figure 2:**
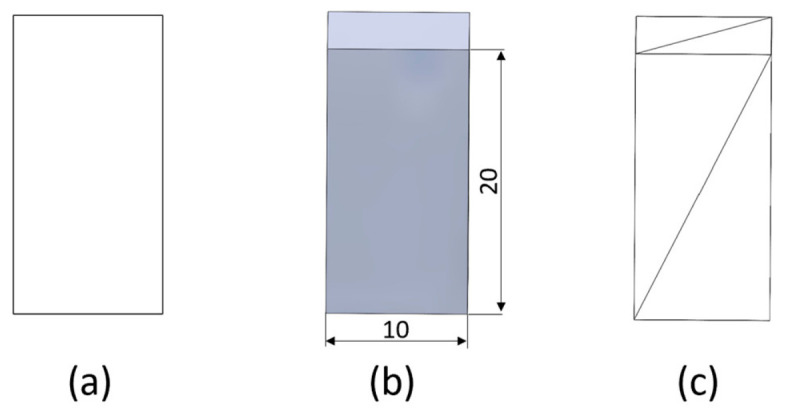
Shape and dimensions of the compression specimen: (**a**) 2D sketch; (**b**) 3D model; (**c**) STL model.

Figure 2 shows the shape and dimensions of the compression specimen.

**Figure 3:**
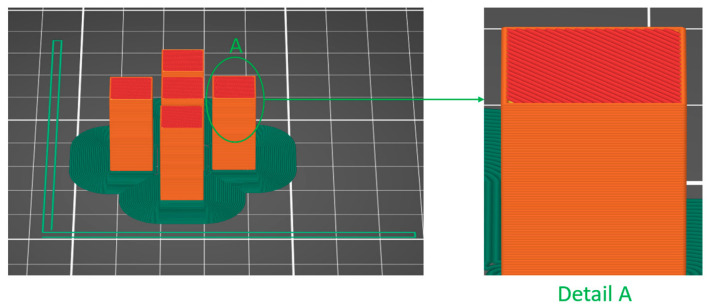
Compression specimens in QIDI Slicer software version 1.2.4.

The STL file was processed by the authors using QIDI Slicer v.1.2.4 software (Figure 3), where the process parameters for thermoplastic extrusion were set (see Table 1), and subsequently the G-Code file was generated for the manufacture of compression specimens from PETG and rPETG [52]. The choice of material is a crucial step; the material must be chosen depending on the type of application for the finished product, as well as its compatibility with the manufacturing equipment used. Polyethylene terephthalate glycol (PETG) is a versatile thermoplastic in applications of additive manufacturing via thermoplastic extrusion in various fields, including aerospace, medical, and automotive, due to its portability and mechanical characteristics [10,53,54,55]. The samples in this study were manufactured using Everfil brand filaments. According to the manufacturer’s data sheet, the filaments have a diameter of 1.75 mm, a diameter tolerance of ±0.02 mm, an ovality tolerance of ±0.015 mm, a thermal conductivity of 0.2 W/m °C, a vicat softening temperature (rate B/50) of 97 °C, a relative temperature index (electrical) of 130 °C, a glass transition temperature of 80 °C and a density of 1.29 g/cm^3^ [56].

Table 1 presents the additive manufacturing parameters for making compression specimens from PETG and rPETG.

**Figure 4:**
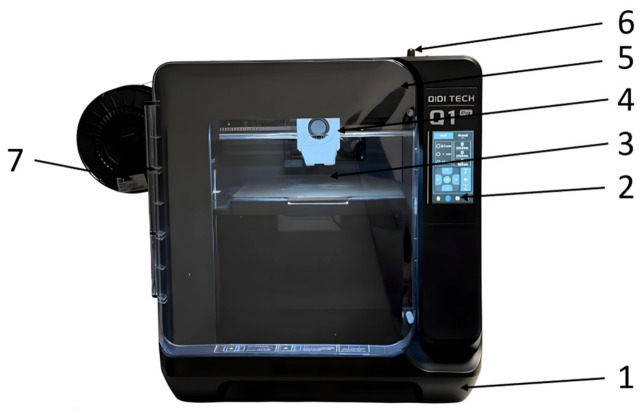
QIDI Q1 Pro printer: 1—frame; 2—capacitive screen for command and control; 3—platform; 4—print head; 5—safety and access door; 6—storage device; 7—filament roll.

Figure 4 shows the printer used for the additive manufacturing of compression specimens by extruding PETG filaments in both virgin and recycled forms.

**Figure 5:**
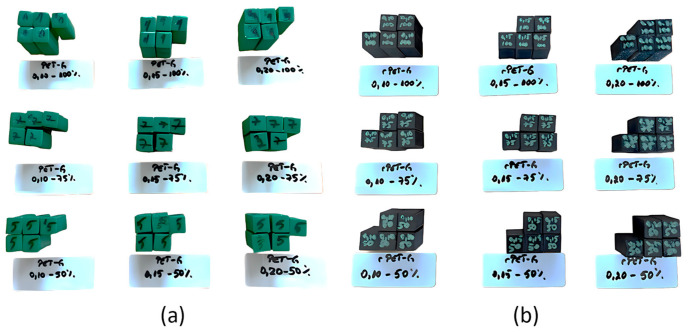
Compression specimens manufactured additively by thermoplastic extrusion on the QIDI Q1 Pro 3D printer: (**a**) PETG; (**b**) rPETG.

The Q1 Pro 3D printer model is manufactured by QIDI. The equipment is equipped with an extruder and stands out due to the temperature control system within the printer enclosure, as well as the automatic leveling system of the printing platform. The dimensions of the printer are 477 × 467 × 489 mm, and its mass is 17 kg. Due to the maximum extruder temperature of 350 °C, the printer supports a wide range of materials, polylactic acid (PLA), acrylonitrile butadiene styrene (ABS), acrylonitrile butadiene styrene reinforced with carbon fibers (ABS-CF), acrylonitrile styrene acrylate (ASA), acrylonitrile styrene acrylate reinforced with carbon fibers (ASA-CF), polyethylene terephthalate glycol (PETG), polyethylene terephthalate glycol reinforced with carbon fibers (PETG-CF), and polyimide (PA). This advantage is important as it offers users a wide range of materials. The printing volume of the printer is 14,406 cm^3^, which is sufficient for most types of work performed on this equipment. Figure 5 shows the compression specimens additively manufactured on the QIDI Q1 Pro 3D printer (Figure 4) by extruding PETG and rPETG filaments.

### 2.2. Performing the Annealing Heat Treatment

**Figure 6:**
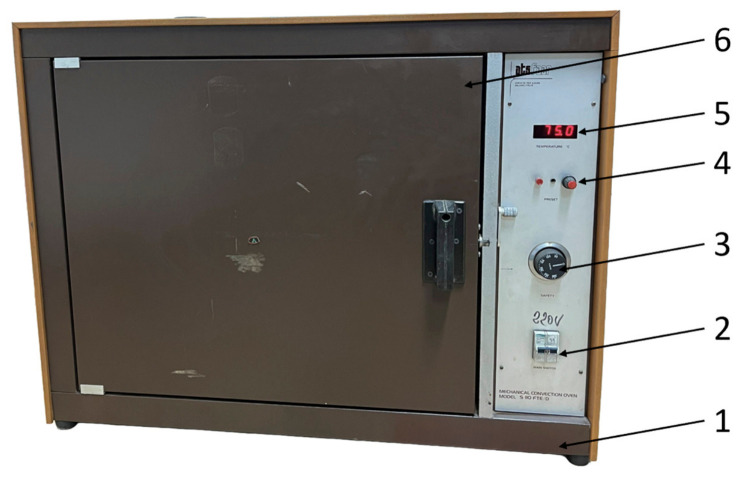
Electric oven for performing thermal annealing treatment on compression specimens additively manufactured from PETG and rPETG: 1—housing; 2—main switch; 3—safety thermostat; 4—temperature control; 5—digital display; 6—safety and access door.

PETG and rPETG compression specimens were subjected to thermal annealing treatment in the ATS FAAR S110 FTE/D electric oven (Figure 6). The following parameters were used to perform the treatment: temperature t = 75 °C and duration d = 180 min. After 180 min, the specimens were slowly cooled. The heat treatment temperature was chosen so that the pieces would not suffer major dimensional changes, and the treatment duration was established following previous research conducted by the authors.

### 2.3. rPETG Compression Testing of Additively Manufactured PETG and rPETG Specimens

**Figure 7:**
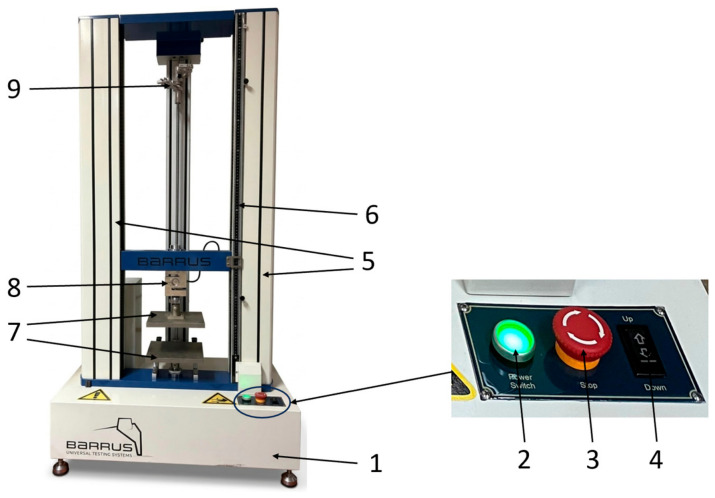
Barrus White 20 kN universal testing machine: 1—frame; 2—start button; 3—emergency stop button; 4—travel adjustment button; 5—columns; 6—gradient ruler; 7—compression testing devices; 8—load cell; 9—extensometer.

In total, 90 compression specimens (45 of PETG and 45 of rPETG) were subjected to compression testing on the Barrus White machine (Figure 7), which has a capacity of 20 kN, using a test speed of 5 mm/min, according to the ISO 604:2002 standard (Plastics—Determination of compressive properties) [57].

## 3. Results

Following the experimental determinations of compression on samples additively manufactured from PETG and rPETG and subsequently heat-treated, 90 experimental data points were obtained, which are summarized in Table 2. 

**Figure 8:**
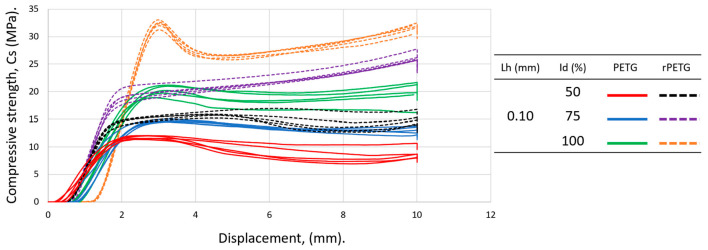
Graphical representation of the compressive strengths of additively manufactured specimens from PETG and rPETG with L_h_ = 0.10 mm and I_d_ = (50–100)%.

Figure 8 contains the graphical representation of the compressive strengths for specimens manufactured with L_h_ = 0.10 mm and I_d_ = (50–100)%.

**Figure 9:**
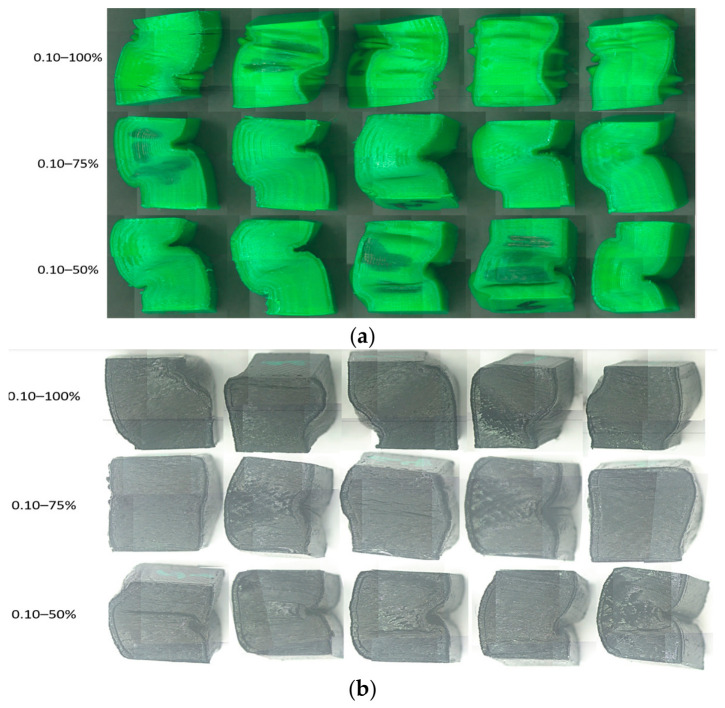
Additively manufactured compression specimens with L_h_ = 0.10 mm and I_d_ = 50/75/100% after performing experimental determinations: (**a**) PETG, (**b**) rPETG.

Analyzing the data summarized in Table 2 and the graph in Figure 8, it can be seen that the average compression performances of rPETG are up to 59.46% higher than those of PETG, regardless of the set value of the percentage filling density used. For I_d_ = 50/75/100%, the compression strengths of the specimens manufactured from rPETG are higher by 35.86/78.92/59.02% compared to those of the specimens manufactured from PETG. In the case of heat-treated rPETG, increasing I_d_ from 50% to 75% resulted in an average improvement in compression strengths of 64.88%, while increasing I_d_ from 75% to 100% led to an average increase of 23.30% in compression strengths. In the case of heat-treated PETG, increasing I_d_ from the minimum value (50%) to the intermediate value of 75% led to a 25.20% increase in average compressive strengths, and increasing I_d_ from 75% to 100% led to an average increase of 38.73% in compressive strengths. Figure 9 shows the samples additively manufactured from PETG filament (Figure 9a) and rPETG (Figure 9b) and heat-treated after performing experimental compression tests.

The morphological analysis of the parts made of PETG (Figure 9a) shows that the specimens collapsed predominantly through lateral buckling, with cracks observed on their surfaces. In the case of the specimens made of rPETG (Figure 9b), it is observed that the specimens collapsed predominantly through barrel-type deformation, and the lack of cracks can be noted.

**Figure 10:**
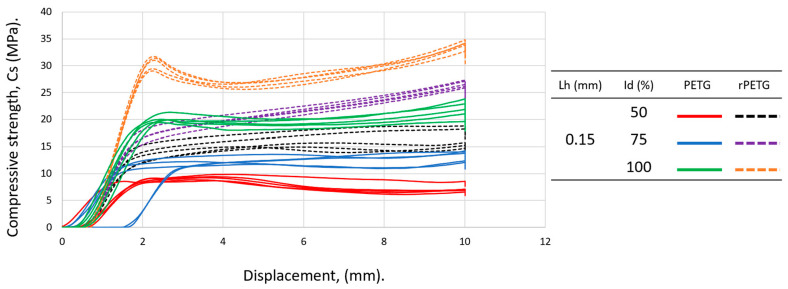
Graphical representation of the compressive strengths of additively manufactured specimens from PETG and rPETG with L_h_ = 0.15 mm and I_d_ = (50–100)%.

Figure 10 contains the graphical representation of the compressive strengths for specimens manufactured with L_h_ = 0.15 mm and I_d_ = (50–100)%. According to the data in Table 2 and the graph in Figure 10, it can be concluded that the compressive strengths of rPETG are 73.78% higher than those obtained for specimens made of PETG. For L_h_ = 0.15 and I_d_ = 50/75/100%, the compressive strengths of specimens made of rPETG are 80.37/99.39/55.33% higher than those of specimens made of PETG. For specimens made of rPETG filament, increasing I_d_ from 50% to 75% resulted in an average compressive strength improvement of 60.24%, while increasing I_d_ from 75% to 100% resulted in a 27.65% increase in the average compressive strengths. For specimens additively manufactured by thermoplastic extrusion of PETG, increasing I_d_ from 50% to 75% led to a 44.96% increase in average compressive strengths, and increasing I_d_ from 75% to 100% led to an average increase of 63.85% in compressive strengths.

**Figure 11:**
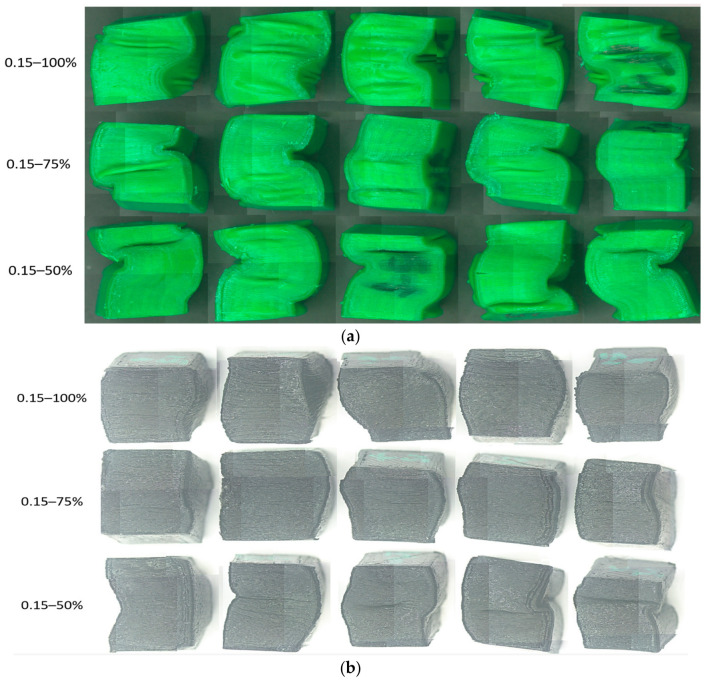
Additively manufactured compression specimens with L_h_ = 0.15 mm and I_d_ = 50/75/100% after performing experimental determinations: (**a**) PETG, (**b**) rPETG.

Figure 11 shows the samples additively manufactured from PETG filament (Figure 11a) and rPETG (Figure 11b) and heat-treated after performing experimental compression tests.

Following the morphological analysis of the parts made of PETG (Figure 11a), it can be seen that the specimens collapsed predominantly due to lateral buckling, with cracks observed on their surfaces, which indicates poor adhesion of the layers. In the case of the specimens made of rPETG (Figure 11b), it can be seen that they collapsed predominantly due to barrel-type deformation, and the absence of cracks indicates superior adhesion of the overlapping layers.

**Figure 12:**
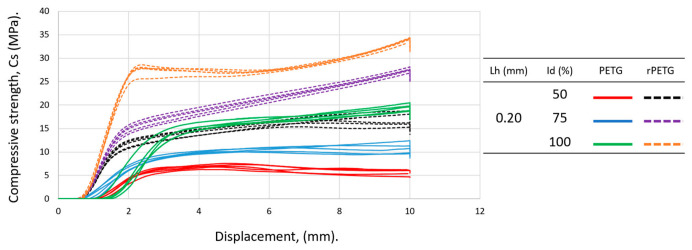
Graphical representation of the compressive strengths of additively manufactured specimens from PETG and rPETG with L_h_ = 0.20 mm and I_d_ = (50–100)%.

Figure 12 contains the graphical representation of the compressive strengths for specimens manufactured with L_h_ = 0.20 mm and I_d_ = (50–100)%. Analyzing the data summarized in Table 2 corresponding to the specimens manufactured with L_h_ = 0.20 mm and I_d_ = 50/75/100%, as well as the graph shown in Figure 12, it can be seen that the average compressive strengths of the specimens manufactured additively from rPETG are 109.85% higher than those of the specimens manufactured from PETG. For I_d_ = 50/75/100%, the compressive strengths of the specimens manufactured from rPETG are 103.92/150.36/74.90% higher than those of the specimens manufactured from PETG. In the case of compression specimens manufactured from rPETG and subsequently heat-treated, increasing the percentage fill density (I_d_) from 50% to 75% led to an average improvement of 61.51% in compressive strengths, and increasing I_d_ from 75% to 100% resulted in a 23.84% increase in average compressive strengths. In the case of compression specimens manufactured from PETG and heat-treated, increasing the percentage fill density (I_d_) from 50% to 75% led to a 57.36% increase in compressive strengths, and increasing I_d_ from 75% to 100% resulted in a 77.27% increase in compressive strengths.

**Figure 13:**
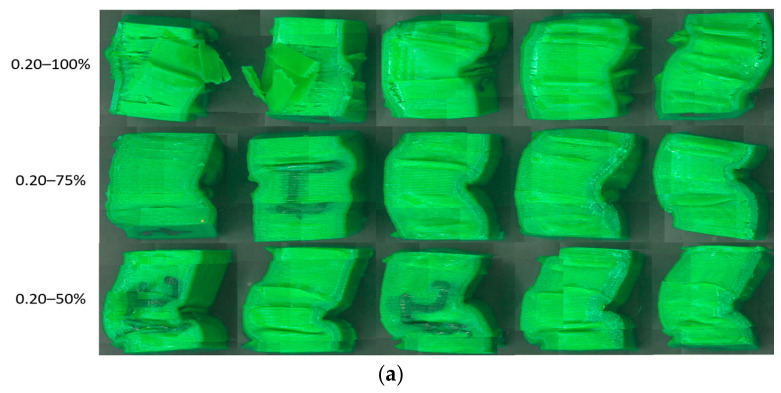

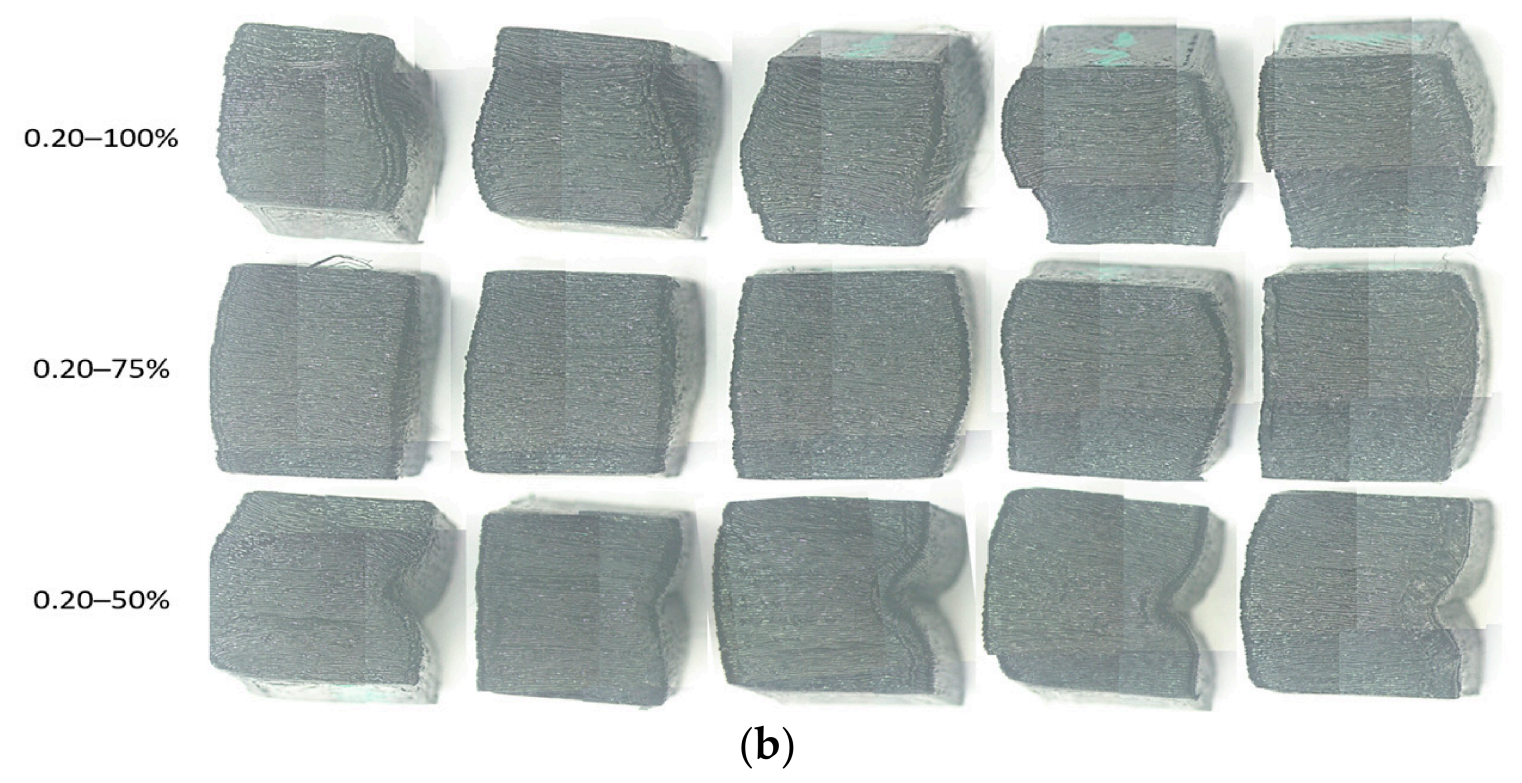
Additively manufactured compression specimens with L_h_ = 0.20 mm and I_d_ = 50/75/100% after performing experimental determinations: (**a**) PETG, (**b**) rPETG.

Figure 13 shows the samples additively manufactured from PETG filament (Figure 13a) and rPETG (Figure 13b) and heat-treated after performing experimental compression tests.

Analyzing Figure 13a, it can be seen that the percentage filling density affects the way the parts fail. In the case of parts manufactured with I_d_ = 100%, delamination and fragmentation of the layers occurred due to the accumulation of internal energy, as well as the height of the layer deposited in one pass (L_h_ = 0.20 mm). In the case of parts manufactured with I_d_ = 75/50%, a lateral buckling failure can be observed. In the case of specimens manufactured from rPETG (Figure 13b), a similar behavior is observed regardless of the value of the percentage filling density (I_d_).

**Figure 14:**
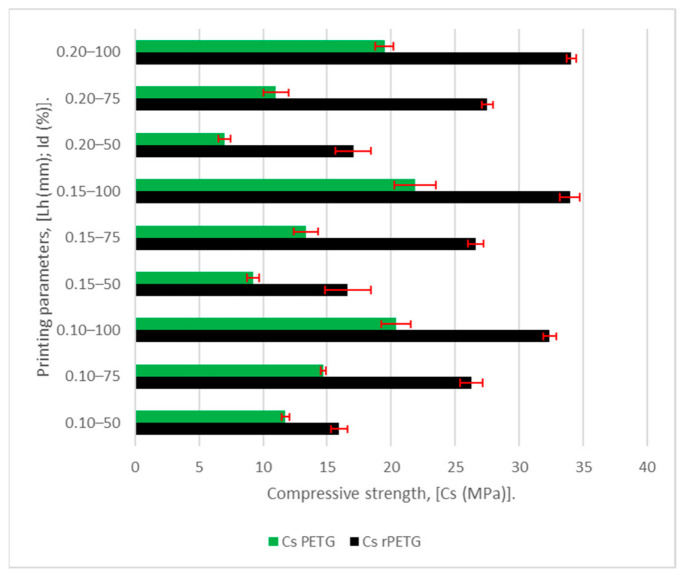
Average compressive strengths (C_s_) of additively manufactured PETG and rPETG specimens.

To analyze the variability of the results obtained from the 90 experimental determinations, the graph in Figure 14 was drawn, showing the average results of the compressive strengths of the two materials (the green and black columns) and the error bars (red symbol).

Analyzing the graph in Figure 14, we observe that the average variation in the compressive strength results of the specimens manufactured from rPETG and PETG is 3.89% and 5.44%, respectively. In the case of rPETG, the variation in compressive strength results corresponding to the specimens manufactured with I_d_ = 50/75/100% is 7.60/2.42/1.65%, and the variation in compressive strength results of the specimens manufactured with L_h_ = 0.10/0/15/0.20 mm is 2.96/5.15/3.57%. In the case of PETG, the variation in compressive strength results corresponding to the specimens manufactured with I_d_ = 50/75/100% is 4.92/5.81/5.60%, and the variation in compressive strength results of the specimens manufactured with L_h_ = 0.10/0/15/0.20 mm is 3.20/6.59/6.54%.

## 4. Evaluation and Optimization of Variable Printing Parameters for the Manufacture of Compression Specimens

As can be seen in Figure 14, the thermoplastic extrusion parameters, specifically the deposited layer height L_h_ = (0.10–0.20) mm and the percentage filling density I_d_ = (50–100)%, have a major influence on the compression specimens manufactured additively from PETG and rPETG. Considering the impact of the manufacturing parameters on the mechanical characteristics, in order to evaluate the influence of the variable manufacturing parameters, ANOVA (analysis of variance) and regression analyses were performed using the Minitab 20.1 software [58].

Table 3 presents the results of the ANOVA analysis for the two materials studied. Table 4 presents the coefficients of the regression model.

Table 5 presents the summaries of the two models corresponding to PETG and rPETG.

Analyzing Table 3, Table 4 and Table 5 we can conclude that the effectiveness of the statistical model is supported by the performance indicators presented in Table 5 [(S—standard deviation of errors; R-sq—R-squared; R-sq (adj)—R-squared adjusted; R-sq (pred)—predicted R squared; PRESS—predicted residual error sum of squares)]. In the case of PETG, all R-squared indicators are above 85%, demonstrating that the model is robust and accurate. In the case of rPETG, the statistical model demonstrates exceptional accuracy, validated by all R-sq values (above 99%).

Following this analysis, regression equations were obtained for each type of material (PETG and rPETG).

(1) $$\begin{array}{cc} Cs\;PETG= & 14.279+1.308\cdot L_{h}\left(0.10\right)\;\mathrm{mm}+0.521\cdot L_{h}\left(0.15\right)\;\mathrm{mm}-1.829\\ & \cdot \;L_{h}\;\left(0.20\right)\;\mathrm{mm}-4.782\cdot I_{d}\;\left(50\right)\%-1.559\cdot I_{d}\;\left(75\right)\%+6.341\\ & \cdot \;I_{d}\;\left(100\right)\% \end{array}$$

(2) $$\begin{array}{cc} \mathrm{Cs}\;r\mathrm{PETG}= & 25.594+0.738\cdot L_{h}\left(0.10\right)\;\mathrm{mm}+0.126\cdot L_{h}\left(0.15\right)\;\mathrm{mm}+0.612\\ & \;\cdot L_{h}\;\left(0.20\right)\;\mathrm{mm}-9.071\cdot I_{d}\;\left(50\right)\%+1.196\cdot I_{d}\;\left(75\right)\%+7.776\\ & \;\cdot I_{d}\;\left(100\right)\% \end{array}$$

**Figure 15:**
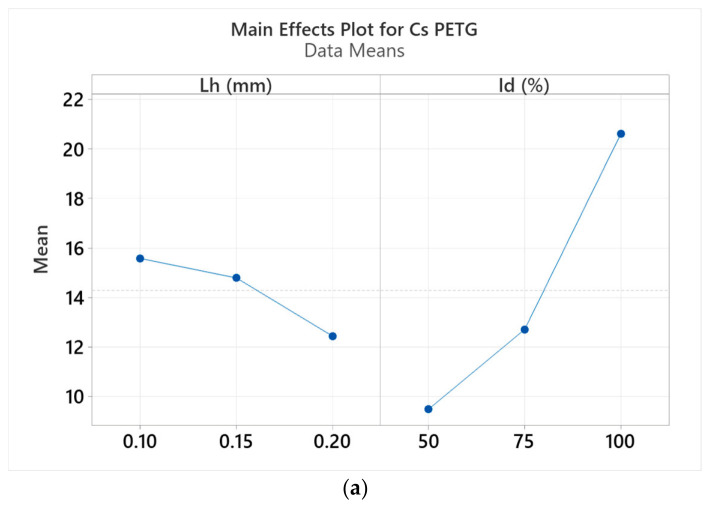

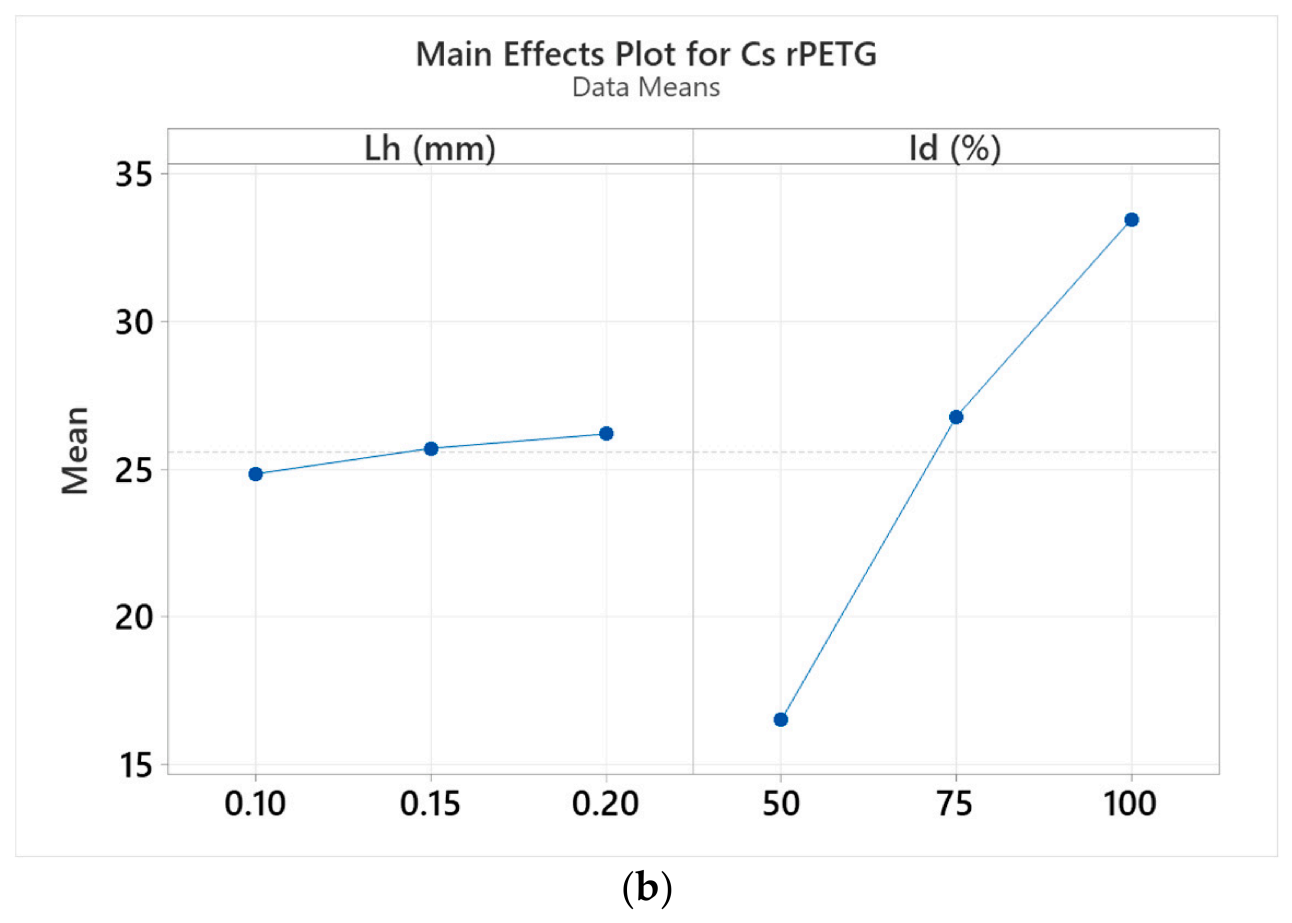
Main effects plot for compressive strength as a function of layer thickness (L_h_) and filling percentage (I_d_): (**a**) PETG; (**b**) rPETG.

Figure 15 presents graphs representing the results of the analysis of the main effects of the variable thermoplastic extrusion parameters (L_h_ and I_d_) on the compression strengths of the specimens manufactured from PETG and rPETG filament.

Following the analysis of the influence of the main effects of the thermoplastic extrusion manufacturing parameters (L_h_ and I_d_) on the compressive strengths of the specimens manufactured from PETG (Figure 15a) and rPETG (Figure 15b), we can conclude that the percentage filling density (I_d_) represents the parameter that crucially influences the mechanical characteristics under compression. Analyzing the two graphs, we observe different behavior of the materials (PETG and rPETG) under compression depending on the height of the layer deposited in one pass (L_h_). In the case of PETG, increasing L_h_ from 0.10 to 0.20 mm led to a 20.09% decrease in compressive strength, and in the case of rPETG, increasing L_h_ from 0.10 to 0.20 mm resulted in a 5.43% increase in compressive strength.

**Figure 16:**
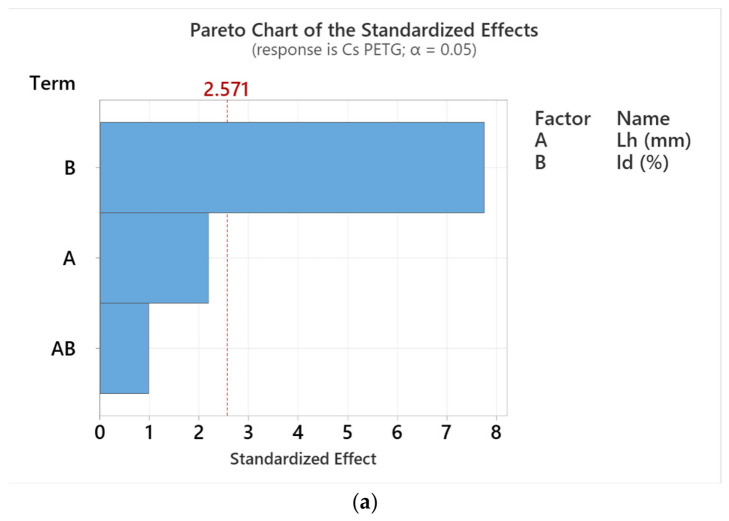

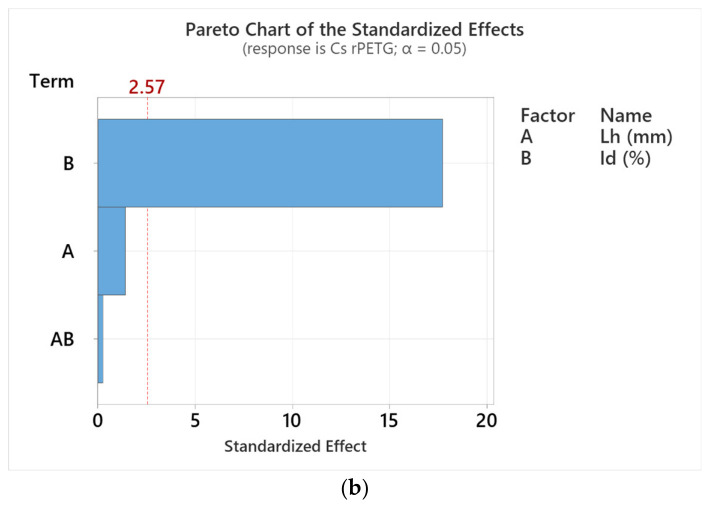
Pareto chart of the standardized effects for compressive strength as a function of layer thickness (L_h_) and filling percentage (I_d_): (**a**) PETG; (**b**) rPETG.

Using Minitab, the design of experiments (DoE) was defined, and subsequently the Analyze Factorial Design function was used to generate the Pareto charts in Figure 16.

The Pareto graphs in Figure 16 show the influence of the variable parameters of thermoplastic extrusion (A = L_h_, B = I_d_ and AB) on the compressive strengths of specimens manufactured from PETG and rPETG. The horizontal blue bars denote the value of the standardized effects, and the vertical red bar represents the critical threshold based on the significance level α = 0.05.

Analyzing the two graphs in Figure 16, we observe that the parameter that significantly influences the compressive strength (C_s_) of the specimens manufactured additively from PETG and rPETG is the percentage filling density (I_d_). In the case of PETG (Figure 16a), the influence of L_h_ on compressive strength is 255.50% lower than the influence of I_d_. The interaction value between Lh and Id (AB) is 0.976 units, which is lower than the I_d_ value by 6.744 units and lower than the L_h_ value by 1.14 units. In the case of specimens manufactured from rPETG filament (Figure 16b), the influence of L_h_ on compressive strength is 1156.03% lower than that of I_d_. The interaction value between L_h_ and I_d_ (AB) is 0.247 units, which is lower than the I_d_ value by 14.463 units and lower than the L_h_ value by 1.163 units.

**Figure 17:**
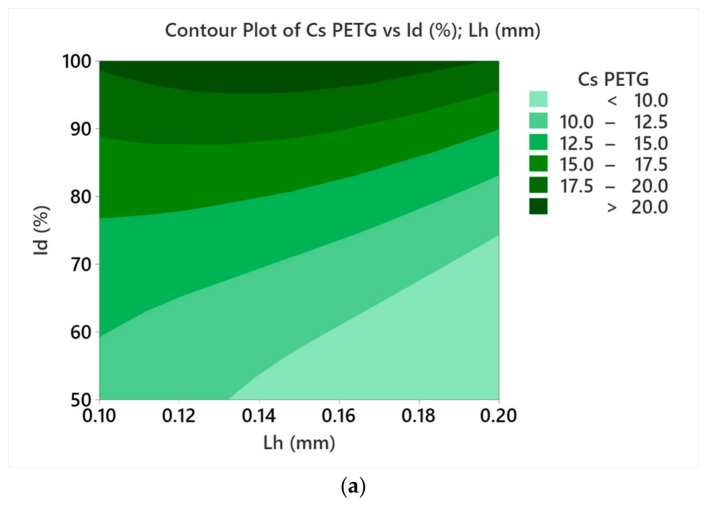

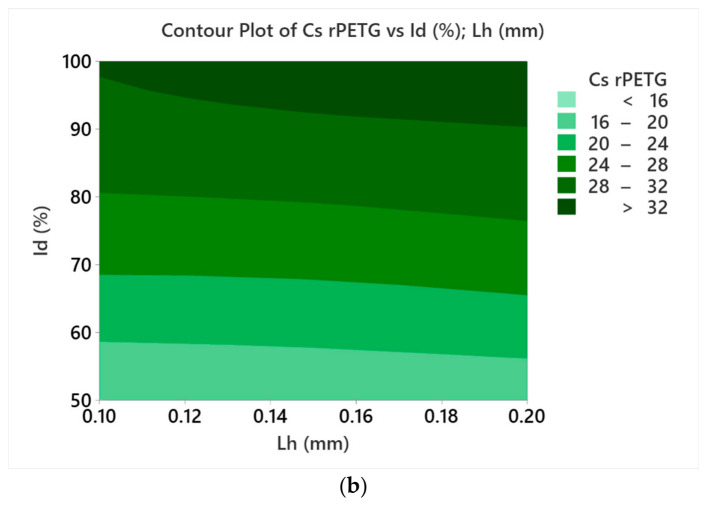
Contour chart for compressive strength as a function of layer thickness (L_h_) and filling percentage (I_d_): (**a**) PETG; (**b**) rPETG.

In Figure 17, the contour graphs illustrate the influence of variable additive manufacturing parameters on the production of compression specimens from PETG and rPETG.

Analyzing the contour plots in Figure 17, we can observe the influence of the two variable parameters of thermoplastic extrusion on the compressive strengths of additively manufactured specimens made from PETG (Figure 17a) and rPETG (Figure 17b). The general conclusion is that the parameter that predominantly influences the compressive strengths is the percentage filling density (I_d_).

**Figure 18:**
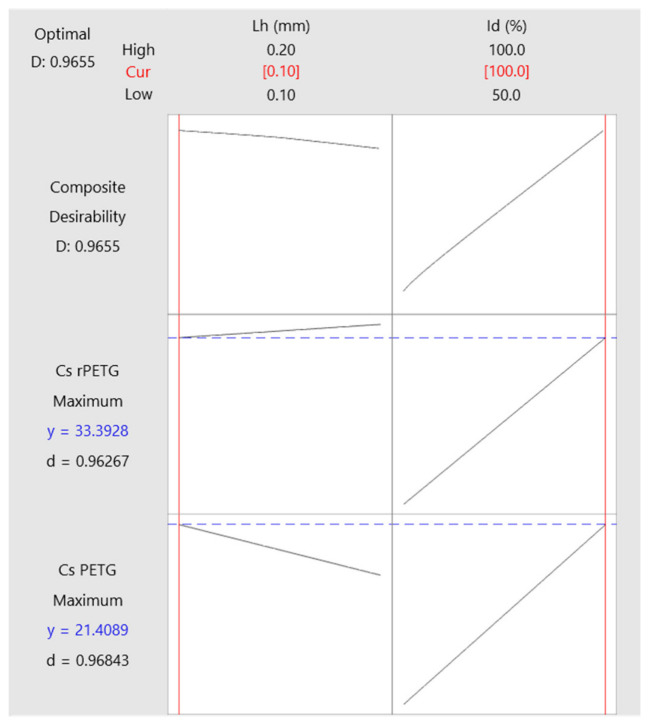
Thermoplastic extrusion parameter optimization graphs for the manufacture of compression specimens from PETG and rPETG.

Figure 18 presents the optimization plots of the variable parameters of thermoplastic extrusion (L_h_ and I_d_) for manufacturing compression specimens from PETG and rPETG. The optimal parameters for manufacturing compression specimens from PETG and rPETG are L_h_ = 0.10 mm and I_d_ = 100%, the desirability value being 0.9655.

## 5. Evaluation of the Influence of Annealing Heat Treatment on the Compressive Strengths of Additively Manufactured PETG and rPETG Specimens

**Figure 19:**
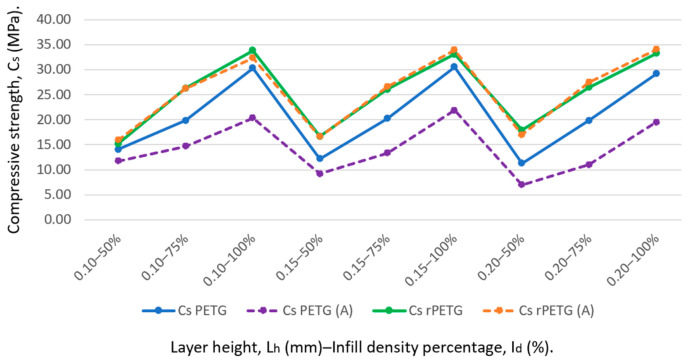
Compressive strength (C_s_) of specimens made from PETG, rPETG and heat-treated PETG, rPETG.

Based on the studies previously published by the authors in [59,60] and the experimental data from this study, a comparative analysis of the compressive strengths of specimens manufactured from PETG (C_s_ PETG) and rPETG (C_s_ rPETG) and subsequently heat-treated [C_s_ PETG (A) and C_s_ rPETG (A)] was conducted. To reduce experimental variability, all specimens were manufactured using the same batch of PETG and rPETG filament, respectively. Figure 19 shows a graph showing comparing the average compressive strengths of specimens manufactured from PETG, rPETG, and those that were subsequently heat-treated.

Analyzing Figure 19, we can see that the samples made of PETG and subsequently heat-treated for 180 min at a temperature of 75 °C obtained compressive strengths significantly lower than the samples that were not heat-treated, the average results of the compressive strengths of the samples made of heat-treated PETG being 31.40% lower than the average compressive strength of the samples made of PETG. Additionally, the average compressive strengths of the samples made of heat-treated PETG are 43.80% lower than those made of rPETG and 44.15% lower than those made of heat-treated PETG. In this case, the application of heat treatment caused molecular relaxation, leading to a decrease in the compressive strengths of PETG.

The average compressive strengths of specimens made from heat-treated rPETG are 0.63% higher than those made from rPETG but not heat-treated. Compared to the compressive strengths of PETG and heat-treated PETG, specimens made from heat-treated rPETG achieved compressive strengths that were 22.85% and 79.07% higher, respectively.

## 6. Conclusions

This study presents the results of research on the influence of thermoplastic extrusion parameters and annealing heat treatment on the compressive strength of specimens manufactured from PETG and recycled PETG. In order to evaluate the influence of the variable parameters of thermoplastic extrusion—the height of the layer deposited in one pass, L_h_ = (0.10; 0.15; 0.20) mm and the percentage filling density, I_d_ = (50; 75; 100)%—a 3^2^-type factorial experimental design was used, which consisted of 9 unique combinations, and for each unique combination, 5 parts were manufactured. In this context, for the study, 90 compression specimens were additively manufactured by thermoplastic extrusion of PETG filaments in both virgin and recycled form (rPETG) using the manufacturing parameters presented in Table 1. The 90 compression specimens were heat-treated using the following parameters: duration d = 180 min and temperature t = 75 °C. The compression specimens additively manufactured by thermoplastic extrusion of PETG and rPETG filaments and subjected to the annealing heat treatment were tested in compression on the Barrus White machine.

The average compressive strength of the 45 specimens manufactured from heat-treated rPETG is 79.09% higher than the compressive strength of the specimens made from heat-treated PETG.

For specimens manufactured from heat-treated PETG, increasing L_h_ from 0.10 to 0.20 mm resulted in a 19.88% decrease in compressive strength, and for specimens manufactured from heat-treated rPETG, increasing L_h_ from 0.10 to 0.20 mm led to a 5.43% increase in compressive strength.

Using Minitab, evaluation and optimization of variable printing parameters for the manufacture of compression specimens was performed. After performing statistical analyses, we can conclude that, of the two variable parameters studied (L_h_ and I_d_), the percentage fill density is the parameter that significantly influences compressive strength.

The annealing heat treatment influenced the compressive properties of the specimens made of PETG and rPETG, as well as their dimensional characteristics (the parts underwent a slight deformation after the heat treatment). In the case of the specimens made of PETG, the annealing heat treatment caused a 31.40% decrease in compressive strength, and in the case of the specimens made of rPETG, the annealing heat treatment caused a 0.63% increase in compressive strength.

For PETG, the annealing heat treatment caused molecular relaxation, which led to a decrease in compressive strength, and in the case of rPETG, a superior thermal stability can be observed, this being generated following the recycling process.

Based on the results obtained in this study, we can conclude that the annealing heat treatment influences the mechanical characteristics of the parts manufactured in compression from PETG and rPETG. Given the results obtained, the authors do not recommend the use of these heat treatment parameters on parts manufactured from PETG and rPETG that are required to be compressed during operation in various industrial applications.

Future research will be oriented in two directions: the first direction represents the analysis of the structures and properties of the materials through scanning electron microscopy (SEM), studying molecular weight distribution (GPC), thermal properties (DSC), rheological behavior (MFI or viscosity) and other intrinsic polymer characteristics. The second direction will consist in applying these heat treatment parameters to other types of specimens (tensile, 3-point bending, impact) made of PETG and rPETG.

## Figures and Tables

**Table 1:** Extrusion parameters used for manufacturing compression specimens from PETG and rPETG.

Category	Parameter	Symbol	Value
Variable parameters	Layer height	L_h_	0.10; 0.15; 0.20 mm
	Infill density percentage	I_d_	50; 75; 100%
Constant parameters	Extrusion temperature	E_t_	250 °C
	Heated platform temperature	B_t_	70 °C
	Infill pattern	I_p_	Grid
	Plate adhesion	P_a_	Brim
	Top fill pattern	T_fp_	Monotonic
	Bottom fill pattern	B_fp_	Monotonic
	Printing speed	P_s_	120 mm/s
	Extrusion width	E_w_	0.42 mm
	Minimum shell thickness	M_st_	1 mm
	Pressure advance	P_ad_	0.056 mm/s
	Seam position	S_p_	Aligned

**Table 2:** Results of experimental compression tests on additively manufactured PETG and rPETG specimens.

Material	Layer Height, L_h_	Infill Density Percentage, I_d_	Compressive Strength, C_s_					Average
-	(mm)	(%)	Sample Number					(MPa)
			1	2	3	4	5	
PETG	0.10	50	11.59	12.01	12.07	11.57	11.38	11.72
		75	15.03	14.53	14.55	14.57	14.71	14.68
		100	21.68	19.74	20.21	18.89	21.29	20.36
	0.15	50	8.66	8.85	9.87	9.22	9.42	9.20
		75	13.79	14.05	14.24	12.39	12.24	13.34
		100	23.83	21.88	22.92	19.62	21.06	21.86
	0.20	50	7.57	6.29	6.79	7.31	6.96	7.57
		75	10.14	10.90	11.39	10.09	12.43	10.14
		100	19.64	18.83	19.79	20.46	18.69	19.64
rPETG	0.10	50	15.79	15.21	15.92	15.77	16.96	15.93
		75	25.71	26.20	27.75	25.72	25.93	26.26
		100	31.62	32.59	32.36	33.05	32.29	32.38
	0.15	50	15.25	18.24	14.95	18.82	15.75	16.60
		75	26.52	26.14	25.89	27.33	27.13	26.60
		100	34.22	34.83	32.70	34.17	33.86	33.96
	0.20	50	16.77	16.21	15.41	18.04	18.76	17.04
		75	26.85	27.56	27.40	27.65	28.12	27.51
		100	34.25	34.24	33.48	34.38	34.02	34.07

**Table 3:** Results of analysis of variance (ANOVA).

Material	Source	Degrees of Freedom (DS)	Sequential Sum of Squares (Seq SS)	Adjusted Sum of Squares (Asj SS)	F-Value	*p*-Value
PETG	L_h_ (mm)	2	15.980	15.980	5.15	0.078
	I_d_ (%)	2	196.528	196.528	63.28	0.001
	Error	4	6.211	6.211	-	-
	Total	8	218.720	-	-	-
rPETG	L_h_ (mm)	2	2.805	2.805	12.64	0.019
	I_d_ (%)	2	437.216	437.216	1970.43	0.000
	Error	4	0.444	0.444	-	-
	Total	8	440.465	-	-	-

**Table 4:** Regression model coefficients.

Material	Parameter	Coefficient	SE Coef	95% CI	*p*-Value
PETG	Constant	14.279	0.415	(13.126; 15.432)	0.000
	L_h_ = 0.10 mm	1.308	0.587	(−0.323; 2.939)	0.090
	L_h_ = 0.15 mm	0.521	0.587	(−1.110; 2.152)	0.425
	L_h_ = 0.20 mm	−1.829	0.587	(−3.460; −0.198)	0.036
	I_d_ = 50%	−4.782	0.587	(−6.413; −3.151)	0.001
	I_d_ = 75%	−1.559	0.587	(−3.190; 0.072)	0.057
	I_d_ = 100%	6.341	0.587	(4.710; 7.972)	0.000
rPETG	Constant	25.594	0.111	(25.286; 25.903)	0.000
	L_h_ = 0.10 mm	−0.738	0.157	(−1.174; −0.302)	0.009
	L_h_ = 0.15 mm	0.126	0.157	(−0.310; 0.562)	0.469
	L_h_ = 0.20 mm	0.612	0.157	(0.176; 1.048)	0.018
	I_d_ = 50%	−9.071	0.157	(−9.507; −8.635)	0.000
	I_d_ = 75%	1.196	0.157	(0.760; 1.632)	0.002
	I_d_ = 100%	7.876	0.157	(7.440; 8.312)	0.000

**Table 5:** Validation parameters of the statistical model.

Material	S	R-sq	R-sq (Adj)	R-sq (Pred)	PRESS
PETG	1.24612	97.16%	94.32%	85.62%	31.444
rPETG	0.333083	99.90%	99.80%	99.49%	2.24663

## Data Availability

Data are contained within the article. The data presented in this study are available upon request from the corresponding author.

## References

[1] Winters K., Jull E.I.L. (2026). The role of FFF process parameters on impact strength: A review. Int. J. Adv. Manuf. Technol..

[2] Fidan I., Naikwadi V., Alkunte S., Mishra R., Tantawi K. (2024). Energy Efficiency in Additive Manufacturing: Condensed Review. Technologies.

[3] May G., Psarommatis F. (2023). Maximizing Energy Efficiency in Additive Manufacturing: A Review and Framework for Future Research. Energies.

[4] Monteiro H., Carmona-Aparicio G., Lei I., Despeisse M. (2022). Energy and material efficiency strategies enabled by metal additive manufacturing—A review for the aeronautic and aerospace sectors. Energy Rep..

[5] Landi D., Zefinetti F.C., Spreafico C., Regazzoni D. (2022). Comparative life cycle assessment of two different manufacturing technologies: Laser additive manufacturing and traditional technique. Procedia CIRP.

[6] Kaikai X., Yadong G., Qiang Z. (2023). Comparison of traditional processing and additive manufacturing technologies in various performance aspects: A review. Arch. Civ. Mech. Eng..

[7] Ötüken R., Alparslan C., Erhan M.F., Bayraktar Ş. (2026). Reinforcement of Novel PLA/17-4 PH Stainless Steel Hybrid Structures Fabricated by FDM: The Effects of Layer Configuration, Infill Density and Pattern. Polymers.

[8] Kantaros A., Katsantoni M., Ganetsos T., Petrescu N. (2025). The Evolution of Thermoplastic Raw Materials in High-Speed FFF/FDM 3D Printing Era: Challenges and Opportunities. Materials.

[9] Menargues S., Navas J., Espinosa I., Baile M.T., Vaz R.F., Picas J.A. (2025). Effect of Additive Manufacturing Parameters on PLA, ABS, and PETG Strength. Processes.

[10] Zisopol D.G., Minescu M., Iacob D.V. (2026). Evaluation and Optimization of Thermoplastic Extrusion Parameters to Improve the Dimensional Accuracy of Additively Manufactured Parts Made of PETG, Recycled PETG, ASA, and Recycled ASA. Polymers.

[11] (2021). Additive Manufacturing—General Principles—Fundamentals and Vocabulary.

[12] Mishra V., Bharat N., Kumar V., Vellaisamy M., Veeman D. (2025). Multi-objective optimization of 3D printed PLA/carbon fibre composite using a combined approach of gray relational analysis and ANOVA. J. Thermoplast. Compos. Mater..

[13] Linul E. (2026). New Insights into Tailoring Anisotropy-Driven Shell Design for Enhanced Compression Performance in Additively Manufactured Structures. Prog. Addit. Manuf..

[14] Marconcini F., Giammarinaro G., Tamburrino F., Caposciutti G., Manzolillo L., Neri P., Paganucci F., Razionale A.V. (2026). Investigation on the material extrusion additive manufacturing of unalloyed magnetic steel. Rapid Prototyp. J..

[15] Aronne M., Schwarzer-Fischer E., Bertana V., Mossotti G., Scheithauer U., Ferrero S., Scaltrito L. (2026). Exploring circularity in ceramic 3D printing: Possibilities and implementation. Open Ceram..

[16] Khuje S., Ku N., Bujanda A., Yu J., Tsang H., Meuse N., Vargas-Gonzalez L., Ren S. (2026). Additive manufacturing pathways for polymer-derived ceramics: Processing, structure, and function. npj Adv. Manuf..

[17] Pires M.V., Pawlikowski G.T., Schultz B., Rountree R., Pasang T., Watanabe M., Misiolek W.Z. (2025). A Comparison of Metal Additive and Micro-metal Additive Manufactured 316L Stainless Steel Produced via Selective Laser Melting, Binder Jetting, and Digital Light Processing. Metall. Mater. Trans. A.

[18] Bauwens T., Corthouts P., De Kock L., Denoiseux B., Ureel M., Coopman R. (2026). Dental Model Analysis in Orthognathic Surgery: Accuracy of 3D Printed FDM and SLA Models in Comparison to Original STL File: An In Vitro Analysis. J. Manuf. Mater. Process..

[19] Iacob D.V., Zisopol D.G., Minescu M. (2024). Technical-Economical Study on the Optimization of FDM Parameters for the Manufacture of PETG and ASA Parts. Polymers.

[20] Zisopol D.G., Minescu M., Iacob D.V. (2025). Evaluation and Optimization of Thermoplastic Extrusion Parameters Influencing the Impact Resistance of Additively Manufactured Samples from PETG and Recycled PETG. Polymers.

[21] Solomon I.J., Sevvel P., Gunasekaran J. (2021). A Review on the Various Processing Parameters in FDM. Mater. Today Proc..

[22] Nicholas P., Rossi G., Eppinger C., Nelson C., Sonne K., Akbari S., Tamke M., Hüls J., O’Connor R., Waschek M. (2026). Design-to-Fabrication Workflows for Large-Scale Continuous FDM Grading of Biopolymer Composites. Appl. Sci..

[23] Ibrahim R.H., Salman H. (2026). Manufacturing of Polymer-Based Composite Material by FDM: Challenges and Opportunities for Functional Parts Design. Next Mater..

[24] Gao W., Zhang Y., Ramanujan D., Ramani K., Chen Y., Williams C.B., Wang C.C.L., Shin Y.C., Zhang S., Zavattieri P.D. (2015). The status, challenges, and future of additive manufacturing in engineering. Comput.-Aided Des..

[25] Ngo T.D., Kashani A., Imbalzano G., Nguyen K.T.Q., Hui D. (2018). Additive Manufacturing (3D Printing): A Review of Materials, Methods, Applications and Challenges. Compos. Part B Eng..

[26] Wach R.A., Wolszczak P., Adamus-Wlodarczyk A. (2018). Enhancement of Mechanical Properties of FDM-PLA Parts via Thermal Annealing. Macromol. Mater. Eng..

[27] Stojković J.R., Turudija R., Vitković N., Górski F., Păcurar A., Pleşa A., Ianoşi-Andreeva-Dimitrova A., Păcurar R. (2023). An Experimental Study on the Impact of Layer Height and Annealing Parameters on the Tensile Strength and Dimensional Accuracy of FDM 3D Printed Parts. Materials.

[28] Martins R.F., Branco R., Martins M., Macek W., Marciniak Z., Silva R., Trindade D., Moura C., Franco M., Malça C. (2024). Mechanical Properties of Additively Manufactured Polymeric Materials—PLA and PETG—For Biomechanical Applications. Polymers.

[29] Hussam H., Soliman M.S., Hassab-Allah I.M., Abdelrhman Y. (2025). Effect of Annealing on FDM-Manufactured ABS Parts. Int. J. Adv. Manuf. Technol..

[30] Szust A., Adamski G. (2022). Using Thermal Annealing and Salt Remelting to Increase Tensile Properties of 3D FDM Prints. Eng. Fail. Anal..

[31] Khan M.U.R., Abas M., Khan A.H., Khan I. (2026). Optimization of Mechanical Properties of Acrylonitrile Styrene Acrylate (ASA) in FDM via TOPSIS-Simulated Annealing Hybrid Approach. Rapid Prototyp. J..

[32] Erdoğan B., Yiğitbaşı E., Hoca İ., Yumuşak G. (2026). Optimization of Tensile Strength in Fused Deposition Modeling-Printed PETG Using Heat Treatment: A Response Surface Methodology Approach. J. Mater. Eng. Perform..

[33] Wang P., Zou B. (2022). Improvement of Heat Treatment Process on Mechanical Properties of FDM 3D-Printed Short- and Continuous-Fiber-Reinforced PEEK Composites. Coatings.

[34] Jayanth N., Jaswanthraj K., Sandeep S., Mallaya N.H., Siddharth S.R. (2021). Effect of Heat Treatment on Mechanical Properties of 3D Printed PLA. J. Mech. Behav. Biomed. Mater..

[35] Jo W., Kwon O.-C., Moon M.-W. (2018). Investigation of influence of heat treatment on mechanical strength of FDM printed 3D objects. Rapid Prototyp. J..

[36] Huang C., Lv D., Zhu Y., Chen G., Chen M., Zhang Y., Han Y., Wu H. (2024). Influences of Heat Treatment on Mechanical Properties of SCF/PEEK Composites in FDM-3D Printing Process with UV Laser Assistance. Polym. Compos..

[37] Nguyen T.K., Lee B.-K. (2018). Post-processing of FDM parts to improve surface and thermal properties. Rapid Prototyp. J..

[38] Chalgham A., Ehrmann A., Wickenkamp I. (2021). Mechanical Properties of FDM Printed PLA Parts before and after Thermal Treatment. Polymers.

[39] Gupta P., Kumari S., Gupta A., Sinha A.K., Jindal P. (2021). Effect of Heat Treatment on Mechanical Properties of 3D Printed Polylactic Acid Parts. Mater. Test..

[40] Akhoundi B., Nabipour M., Hajami F., Shakoori D. (2020). An Experimental Study of Nozzle Temperature and Heat Treatment (Annealing) Effects on Mechanical Properties of High-Temperature Polylactic Acid in Fused Deposition Modeling. Polym. Eng. Sci..

[41] Jayswal A., Adanur S. (2022). Effect of Heat Treatment on Crystallinity and Mechanical Properties of Flexible Structures 3D Printed with Fused Deposition Modeling. J. Ind. Text..

[42] Al Rashid A., Koç M. (2025). 3D-Printed Recycled Polyethylene Terephthalate (PET) Sandwich Structures—Influence of Infill Design and Density on Tensile, Dynamic Mechanical, and Creep Response. Int. J. Lightweight Mater. Manuf..

[43] Al Rashid A., Koç M. (2023). Additive Manufacturing for Sustainability and Circular Economy: Needs, Challenges, and Opportunities for 3D Printing of Recycled Polymeric Waste. Mater. Today Sustain..

[44] Alka T.A., Raman R., Suresh M. (2024). Research Trends in Innovation Ecosystem and Circular Economy. Discov. Sustain..

[45] Woern A.L., Byard D.J., Oakley R.B., Fiedler M.J., Snabes S.L., Pearce J.M. (2018). Fused Particle Fabrication 3-D Printing: Recycled Materials’ Optimization and Mechanical Properties. Materials.

[46] Zisopol D.G., Minescu M., Iacob D.V. (2025). A Study on the Optimization of FDM Parameters for the Manufacturing of Compression Specimens from recycled ASA in the Context of the Transition to the Circular Economy. Eng. Technol. Appl. Sci. Res..

[47] Chaudhary G., Ghag N. (2026). Unlocking AI-driven innovations in textile design for a circular economy. Des. J..

[48] Leong W.Y. (2025). Circular Economy Through Green Additive Manufacturing in Medical Device Manufacturing. Eng. Proc..

[49] Das T.K., Jesionek M., Ali M.D., Ganguly S., Poater A. (2026). Advancing sustainability: Circular economy strategies for fibrous polymer composites. Clean. Mater..

[50] (2023). Vosviewer.

[51] (2023). The Solution for 3D CAD, Design and Product Development. https://www.solidworks.com/.

[52] (2025). 3D Printer Software & Firmware. QIDI Slicer.

[53] Yan C., Kleiner C., Tabigue A., Shah V., Sacks G., Shah D., DeStefano V. (2024). PETG: Applications in Modern Medicine. Eng. Regen..

[54] Valvez S., Silva A.P., Reis P.N.B. (2022). Compressive Behaviour of 3D-Printed PETG Composites. Aerospace.

[55] Tzotzis A., Nedelcu D., Mazurchevici S.-N., Kyratsis P. (2024). Surface Quality Evaluation of 3D-Printed Carbon-Fiber-Reinforced PETG Polymer During Turning: Experimental Analysis, ANN Modeling and Optimization. Polymers.

[56] Filament Everfil PETG S 10 Diam/1.75 mm, Colour/Green, Weight/1.00kg Net. https://kordo.eu/en/product/filament-everfil-petg-s10-sred-175mm-kolor-perl-green-ral6018-waga-100kg-netto/.

[57] (2002). Plastics—Determination of Compressive Properties.

[58] (2021). Improve Process and Quality with Data-Driven Analysis. Minitab.

[59] Zisopol D.G., Minescu M., Iacob D.V. (2024). A Study on the Influence of FDM Parameters on the Compressive Behavior of PET-G Parts. Eng. Technol. Appl. Sci. Res..

[60] Zisopol D.G., Minescu M., Iacob D.V. (2024). A Study of the Optimization of FDM Parameters for the Manufacture of Compression Specimens from Recycled PETG in the Context of the Transition to the Circular Economy. Eng. Technol. Appl. Sci. Res..

